# Intraoperative ipsilateral subclavian port catheter implantation in resectable breast cancer patients: A novel, safe, and convenient clinical practice

**DOI:** 10.1002/cam4.3595

**Published:** 2020-11-04

**Authors:** Feng Ye, Yubo Liu, Ping Yu, Na Li, Yan Wang, Xiaoming Xie, Jun Tang

**Affiliations:** ^1^ Department of Breast Oncology Sun Yat‐Sen University Cancer Center State Key Laboratory of Oncology in South China Collaborative Innovation Center for Cancer Medicine Guangzhou Guangdong China; ^2^ Department of Ultrasound Sun Yat‐Sen University Cancer Center State Key Laboratory of Oncology in South China Collaborative Innovation Center for Cancer Medicine Guangzhou Guangdong China

**Keywords:** breast cancer, chemotherapy, clinical practice, intra‐operation ipsilateral implantation, port catheter

## Abstract

**Background:**

Port catheter (PC) is a classical route of administering chemotherapy for breast cancer patients. We established a standard operating procedure (SOP) of intraoperative ipsilateral subclavian PC implantation in selected resectable breast cancer patients.

**Methods:**

We conducted a prospective clinical study to assess its safety and complications. A total of seventy six resectable breast cancer patients were included for intraoperative ipsilateral subclavian PC implantation. Thirty patients receiving conventional percutaneous contralateral PC implantation under local anesthesia at the same period were recruited as control group. The time consuming of implantation, and PC‐related complications were recorded. Visual analog scale questionnaires were used to assess patients’ satisfaction.

**Results:**

Compared with conventional contralateral PC implantation under local anesthesia, SOP for intraoperative ipsilateral subclavian PC implantation significantly shortens the time consuming (11.6 vs. 28.6 min, *p *< 0.001). With a median retention time of 6.3 months, the overall incidence rate of PC‐related complications is 21%, of which the most common complications are infections and venous thromboembolism (7.9% for each). Most patients (86.8%) with intraoperative ipsilateral subclavian PC implantation have completed the whole chemotherapy successfully. Due to the general anesthesia and shorter time consuming, intraoperative implantation gains significantly more patients' satisfaction.

**Conclusions:**

In the present study, we develop a SOP for intraoperative ipsilateral subclavian PC implantation in resectable breast cancer patients, which is noval, convenient, and safe. In selected breast cancer patients with indications for adjuvant chemotherapy, this practice could significantly shorten the time consuming of PC implantation and improve the degree of patients' satisfaction.

## Graphical abstract

In the present study, we established a standard operating procedure (SOP) of intraoperative ipsilateral subclavian PC implantation and conducted a prospective study in selected resectable breast cancer patients to assess its safety and complications. Compared with conventional contralateral PC implantation under local anaesthesia, SOP for intraoperative ipsilateral subclavian PC implantation significantly shortens the time consuming (11.6 vs. 28.6 min, *p* < 0.001). Due to the general anesthesia and shorter time consuming, intraoperative implantation gains significantly more favors in all aspects of patients’ satisfaction assessment. With a median retention time of 6.3 months, the overall incidence rate of PC‐related complications is acceptable, of which the most common complications are infections and venous thromboembolism.

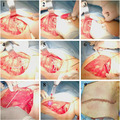

## 1 BACKGROUND

Breast cancer is the most common female malignancy worldwide, with about 260 thousands new incidence and 40 thousands deaths per year in America.^1^ Adjuvant chemotherapy plays an important role in treatments for early‐stage invasive breast cancer, along with surgery, radiotherapy, endocrine therapy, and anti‐HER2 therapy.^2^, ^3^ It is frequently proposed in cases of poor prognosis factors: tumor size > 2 cm, LN positive, high histological grade, vessel invasion, unsatisfactory molecular subtypes (ie, ER/PR negative, HER‐2+, triple negative breast cancer [TNBC]), and high Ki67 index.^4^ Although genotyping methods, that is, Oncotype X, MammaPrint test, etc., have exclude some patients with low recurrence risk from chemotherapy,^5^ it is estimated that about 70% of operative breast cancer patients benefit from adjuvant chemotherapy.^6^ The common adjuvant chemotherapy regimen for breast cancer is based on 4–8 cycles (3–6 months); in case of HER‐2 positive, the adjuvant therapy would prolong to 12 months. Anthracycline, cyclophosphamide, paclitaxel, and carboplatin are the major cytotoxic drugs used.^2^, ^4^ The side effects of these drugs include myelosuppression, vomit, alopecia, and venous toxicities,^7^ etc.

A central venous device is normally required for the administration of adjuvant chemotherapy to avoid peripheral venous punctures and venous toxicities.^8^ To date, the peripherally inserted central catheter (PICC) and Port catheter (PC) have been the major two standard devices used in this indication.^9^, ^10^ PICC can be easily inserted and removed, but it needs maintenance at least once a week. PC is another classical route of administering chemotherapy, which provides deep venous access and allows for iterative perfusions, even for years.^11^ The same pattern of complications (mainly thrombus events^12^ and infections^13^) is observed for both PC and PICC. Compared with PICC, PC has been proved to be associated with lower risk of major complications (infection, thrombus, etc.) and costs.^14^, ^15^

However, the insertion and removal of a PC are more invasive than PICC. Conventionally, a PC would be implanted percutaneously through the contralateral subclavian or internal jugular vein under local anesthesia a couple of days later after breast operation.^16^, ^17^ The subclavian vein has been the prior approach for PC implantation in our institute. The subclavian vein approach for PC implantation would need the guide of ultrasound.^18^, ^19^ Nevertheless, it perhaps still needs several times of puncture when subclavian vein is too thin or has anatomical variations (two‐branch subclavian, reversal position of artery and vein, etc.). The anxiety and mental tension of patients, pain of invasive practice, and difficulty in puncture have been the major problems in a PC implantation, which could make it consuming hours, or finally switch to general anesthesia or jugular vein approach. These embarrassing problems significantly decrease the patients’ satisfaction physically and psychologically.^16^, ^20^, ^21^ Thus, we aim to develop a more convenient and comfortable method for PC implantation to increase patients’ experience.

In a previous study, Omer etc. demonstrated that a PC could be implanted on either ipsilateral or contralateral side of the operation in breast patients suffering mastectomy and axillary lymph node dissection (ALND), with no statistical difference in PC‐related complications.^22^ Since the ipsilateral axillary/subclavian vessels could be clearly anatomized and exposed during mastectomy and ALND, we seek to explore whether a port catheter could be implanted through ipsilateral axillary/subclavian vein intra‐operatively. Here, we established a standard operating procedure (SOP) of intraoperative ipsilateral axillary/subclavian port catheter implantation and conducted a prospective clinical study in selected resectable breast cancer patients to assess its safety and complications.

## 2 PATIENTS AND METHODS

### 2.1 Study design

This prospective study was conducted at our institute. All patients were treated between April 2015 and May 2017. A total of 76 patients were included. The choice of PC implantation approach was at the discretion of surgeons. The inclusion criteria were listed as follows: (a) Female patients aged 18–70; (b) Initial treatment with diagnose of unilateral invasive breast cancer; (c) Undergo mastectomy and ALND; (d) Have indications for chemotherapy according to pre‐operative assessment (with one or more poor prognostic factors): Tumor size >2 cm, LN positive proved by pathology (fine needle or core needle biopsy), unsatisfactory molecular subtypes (ie, ER/PR negative, HER‐2 positive, TNBC), high histological grade (WHO Ⅲ), lymphovascular invasion, high Ki67 index (>20%), young onset age (<35); (e) axillary/subclavian vein diameter ≥5 mm. We excluded patients with a history of thoracic radiation therapy, bilateral axillary node dissection, anticoagulant therapy at cancer diagnosis, thrombosis, renal dysfunction, chemotherapy contraindication, pregnancy or breast‐feeding, and psychiatric disease. All patients accepted adjuvant chemotherapy using PC after breast operation, depending on TNM stage and molecular subtype.

We also selected 30 patients receiving percutaneous conventional contralateral PC implantation under local anesthesia at the same period as control group. Selection method: we selected the first two patients receiving conventional contralateral PC implantation of each month from May 2015 to July 2016. The inclusion and exclusion criteria were generally the same as mentioned above.

The characteristics of the included patients are listed in Table 1.

**Table 1:** Characteristics of breast cancer patients receiving subclavian port catheter implantation

Characteristic	Ipsilateral patients, *n* = 76 (100%)	Contralateral patients, *n* = 30 (100%)	Characteristic	Ipsilateral patients, *n* = 76 (100%)	Contralateral patients, *n* = 30 (100%)
**Age (years)**			**LVI**		
Median	46.3	41.5	No	42 (55.3)	15 (50.0)
≦40	23 (30.3)	15 (50.0)	Yes	34 (44.7)	15 (50.0)
41–60	45 (59.2)	14 (46.7)	**PNI**		
≧60	8 (10.5)	1 (3.3)	No	62 (81.6)	25 (83.3)
**Menopausal**			Yes	14 (18.4)	5 (16.7)
No	55 (72.4)	24 (80.0)	**ER**		
Yes	21 (27.6)	6 (20.0)	Negative	17 (22.4)	10 (33.3)
**Histologic grade**			Positive	59 (77.6)	20 (66.7)
G1	1 (1.3)	0 (0.0)	**PR**		
G2	41 (54.0)	16 (53.3)	Negative	24 (31.6)	13 (43.3)
G3	34 (44.7)	14 (46.7)	Positive	52 (68.4)	17 (56.7)
**T stage**			**HER−2**		
T1	30 (39.4)	9 (30.0)	Negative	51 (67.1)	14 (46.7)
T2	43 (56.6)	15 (50.0)	Positive	25 (32.9)	16 (53.3)
T3	2 (2.7)	2 (6.7)	**Ki67 index**		
T4	1 (1.3)	4 (13.3)	<20	25 (32.9)	7 (23.3)
***N* stage**			≧20	51 (67.1)	23 (76.7)
N0	32 (42.1)	10 (33.4)	**Chemotherapy**		
N1	24 (31.6)	9 (30.0)	AC or TC	18 (23.7)	5 (16.7)
N2	12 (15.8)	4 (13.3)	AC‐T	58 (76.3)	25 (83.3)
N3	8 (10.5)	7 (23.3)	**Anti‐HER2 therapy**		
**TNM stage**			No	63 (82.9)	18 (60.0)
I	18 (23.7)	18 (23.3)	Yes	13 (17.1)	12 (40.0)
II	37 (48.7)	11 (36.7)	**Radiotherapy**		
III	21 (27.6)	12 (40.0)	No	41 (53.9)	12 (40.0)
			Yes	35 (46.1)	18 (60.0)

Note

Ipsilateral patients: Intraoperative ipsilateral subclavian port catheter implantation; Contralateral patients: Conventional percutaneous contralateral subclavian port catheter implantation.

Abbreviations: HER‐2, Human epidermal growth factor receptor‐2; HR, Hormone receptor.

All participants have signed informed consent and this study was approved by our Ethics Committee.

### 2.2 Surgical technique

**Figure 1:**
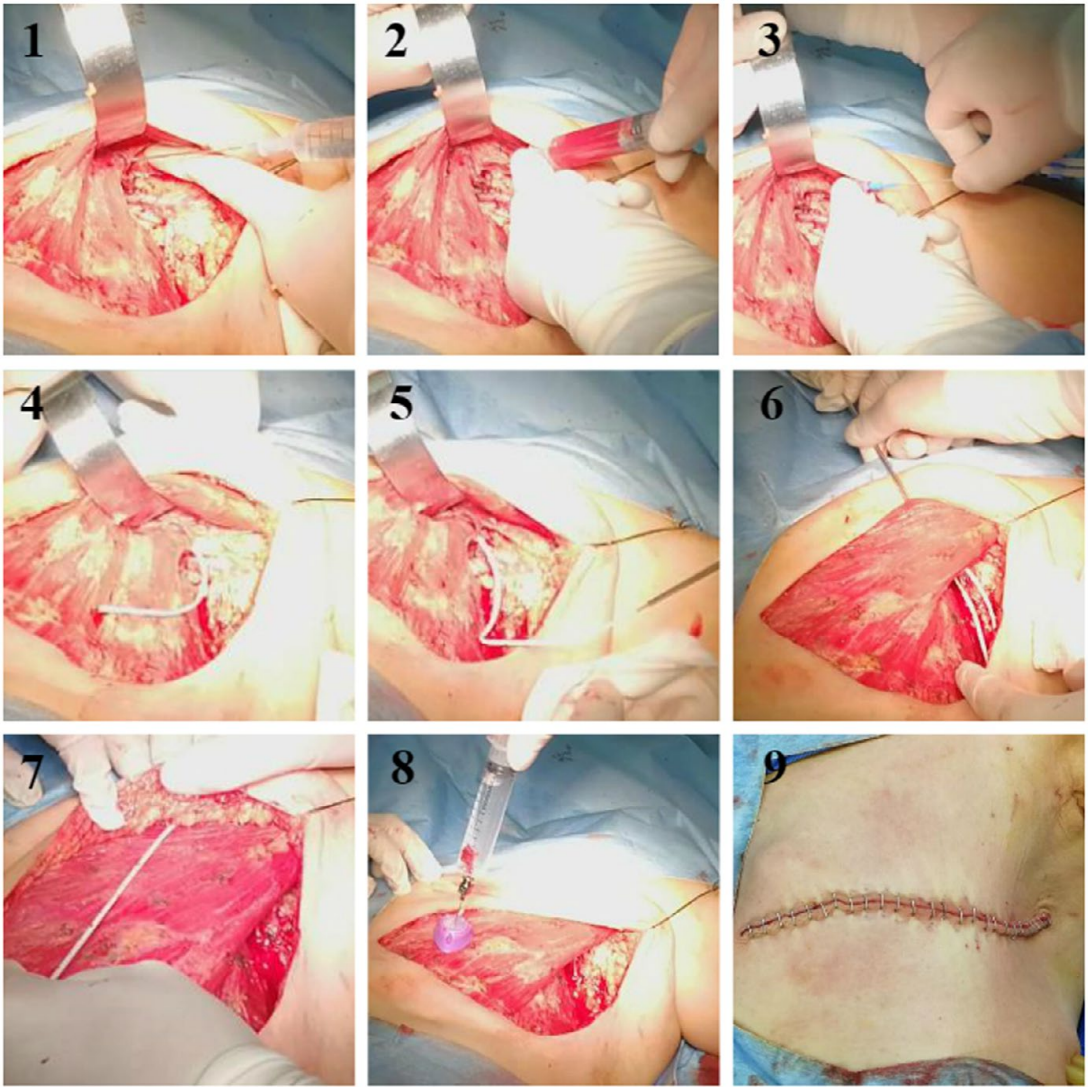
Standard operating procedure for an intraoperative ipsilateral axillary/subclavian PC implantation

We developed a SOP for an intraoperative ipsilateral axillary/subclavian PC implantation:

The patient is postured by supine position abducting the upper limb by 90° under general anesthesia. After standardized modified radical mastectomy, the ipsilateral axillary/subclavian vessels are well exposed. A port catheter and accessories are prepared. Surgeons do the axillary/subclavian vein (level Ⅱ/Ⅲ) puncture under direct vision. A guide wire is put in the vein through the induction syringe. Then, the induction syringe is removed and the dilater is put in. The guide wire is removed and the catheter is put in axillary vein to a suitable depth (usually 15–20 cm according to the height of patient). The catheter is passed through the Pectoralis major to decrease the flexibility. The catheter is cut at an appropriate length (usually 25–30 cm). Finally, surgeons link the catheter to the transfusion port and fix the port on pectoralis major with suture. After the device is tested, surgeons seal up the port, indwell the drainage tubes and close the wound (see Figure 1; Video S1). Time consuming of implantation is recorded (from puncture to wound closure).

Standard operating procedure (SOP) for a B‐ultrasonic guided percutaneous conventional contralateral subclavian PC implantation has been reported in many studies.^23^, ^24^ In the present study, we followed the previous procedures (see the instructions and the sketch map in additional file 2: Supplementary File 1). Time consuming of implantation is recorded (from puncture to wound closure, as indicated in the instructions), too.

### 2.3 Follow‐up and complications related to PC

All of the patients in the present study received chemotherapy and anti‐HER2 therapy (if needed) after operation. Patients with PCs were checked every time of injection. Doppler‐ultrasonography (DUS) was used to detect the venous thromboembolism (VTE) in the ipsilateral upper limb, neck, or head (pain, edema, or headache) every 3 months.

Complications related to the PCs are sorted as follows: puncture complications (pneumothorax on pulmonary X‐ray, hematoma, arterial puncture, nerve damage, puncture failure), infections (local infection (redness and swelling, pain, and even abscess), or catheter‐related bacteremia (fever, shock)), VTE on the catheter confirmed by DUS, infusion extravasation and mechanical complications (catheter dislodgement or migration, reversal of the port, occlusion despite the use of heparin, or urokinase protocols). We recorded the complications observed between the device insertion and its removal.

### 2.4 Removal of PCs

PCs were removed under the situations of severe complications, such like uncontrolled local infection or catheter‐related bacteremia/septicemia, VTE, catheter dislodgement from the vein. For other patients with no severe complications, PC were removed after the end of the all cycles of chemotherapy (or anti‐HER2 therapy if needed) and check‐up (mostly at 6 or 12 months from the implantation).

### 2.5 Patient's satisfaction assessment

For the purpose of evaluation, the visual analog scale (VAS) questionnaires were used, as described in a previous study.^16^ Using the VAS, patients evaluated their sensation of pain (0 = no pain; 10 = extreme pain), their level of tension during the procedure(0 = no tension; 10 = extreme tension), their satisfaction with the procedure (0 = highly dissatisfied; 10 = highly satisfied), on implantation of PC.

### 2.6 Statistical analysis

Comparisons between the two groups of patients were performed using the *T* test. The statistical analyses were performed by the SPSS, version 22.0 (SPSS Inc.). A tow‐side *p* < 0.05 was thought to be statistically significant.

## 3 RESULTS

### 3.1 Intraoperative ipsilateral subclavian PC implantation is a convenient approach

We recorded the time consumption of PC implantation, and the statistics revealed the mean time of an intraoperative ipsilateral subclavian PC implantation was 11.6 min (range from 8 to 15 min). We also chose 30 patients receiving conventional contralateral PC implantation in the same period at random, and recorded the consuming time. It took a mean 28.6 min (range from 22 to 40 min) for a percutaneous conventional contralateral subclavian PC. Intraoperative PC implantation significantly shortens the time (*p *< 0.001).

### 3.2 Complications related to intraoperative ipsilateral axillary PC implantation

We record the time for removing drainage tubes and the complications related to PC implantation in the follow‐up. The criteria for removing drainage tubes is <15 ml/24 h for one tube in our institute. The mean time for removing drainage tubes is 7.05 days, and for most patients, drainage tubes can be removed within one week after operation, which is comparable to those 30 patients mentioned above without intraoperative ipsilateral axillary PC implantation (mean time for removing drainage tubes is 6.46 days).

The median retention time for patients with the intraoperative ipsilateral axillary PC is 6.3 months (about 190 days), of which PC was retained for 6 months in 56 patients, 12 months in 9 patients, 15 months in 1 patients, <6 months in 10 patients.

As to complications, due to the puncture of vessels under direct eyesight, severe puncture‐related complications (hemathorax, pneumothorax, and puncture of artery) have be efficiently avoided in our study. As shown in Table 2, the overall rate of patients with major complications is approximately 17.1%. Here we list and describe the major complications.

**Table 2:** PC‐related complications and indications for removal

	Patients, *n* (%)
Total	76 (100)
Patients with complications	13 (17.1)
Overall complications	16 (21.0)
Reason for removal	
Complication	10 (13.2)
End of chemotherapy	66 (86.8)
Type of complications	
Hematoma	1 (1.3)
Catheter dislodgement	4 (5.3)
Infections	6 (7.9)
VTE	6 (7.9)
Leakage	0 (0)

Note

Three patients suffered Infections and VTE concurrently.

#### 3.2.1 Infections

**Figure 2:**
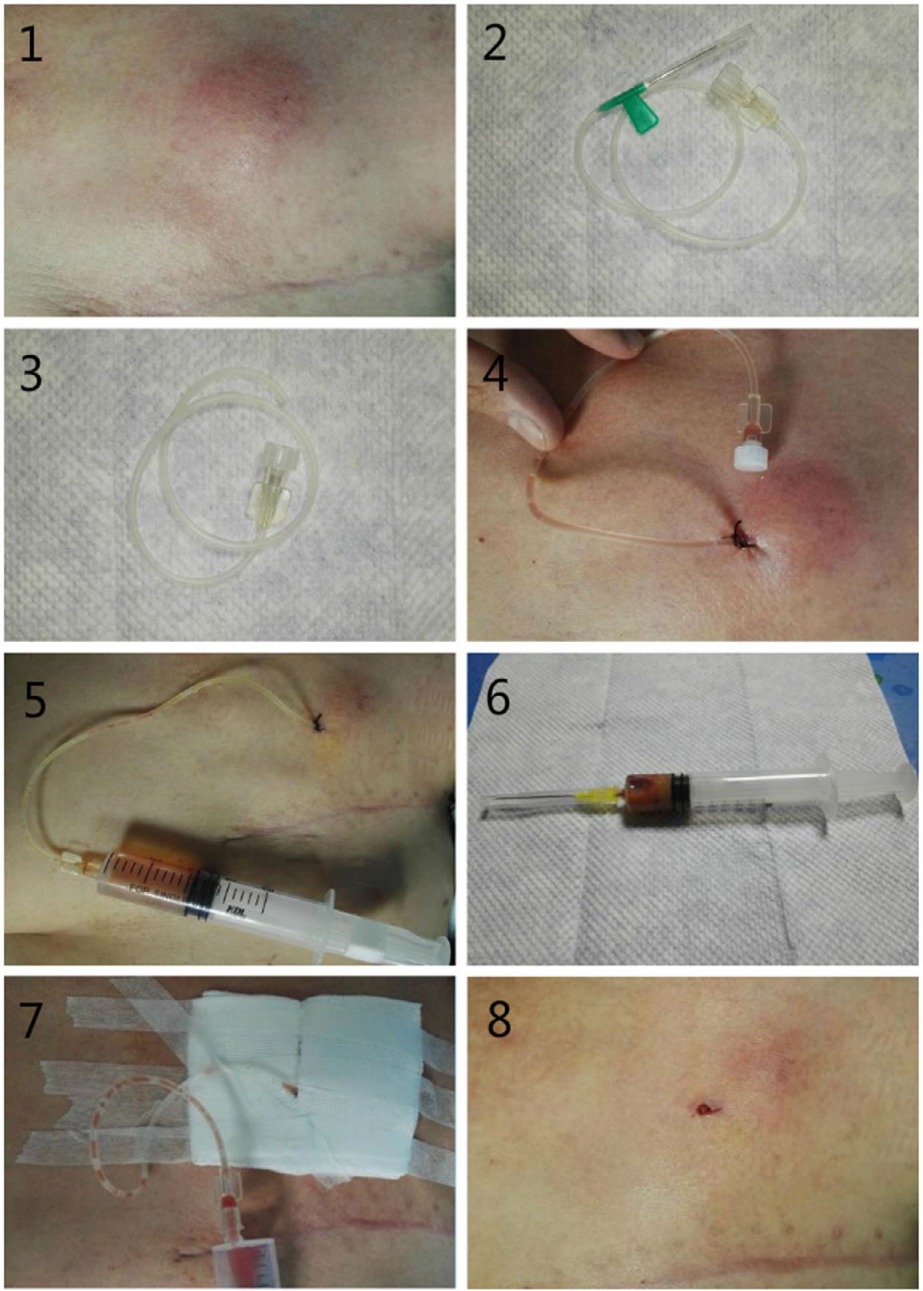
A tiny lavage and drainage device using scalp needle and syringe for local infections, especially effective for abscess

In our study, six patients (6/76, 7.9%) suffered from PC‐related infections. Antibiotic therapy is the most important treatments. For those patients with local abscess around the port, we have invent a tiny lavage and drainage device using scalp needle and syringe (see Figure 2). This tiny lavage and drainage device is effective for local infection and could keep patients from PC removal. If the infection could not be controlled and even develop into septicemia, the PC would be removed. In our study, three of the six infected patients (3/6) have also developed venous thromboembolism concurrently.

#### 3.2.2 Venous thromboembolism (VTE)

VTE is also a common complication for PCs. In our study, six patients (6/76, 7.9%) suffered from VTE in the ipsilateral upper limb, axillary, or neck. The symptoms of VTE are edema of the ipsilateral upper limb, pain, or fever. As mentioned above, VTE could be complicated with infections. Of the six patients with VTE, three patients (3/6) got infections (bacteremia) at the same time.

#### 3.2.3 Catheter dislodgement

**Figure 3:**
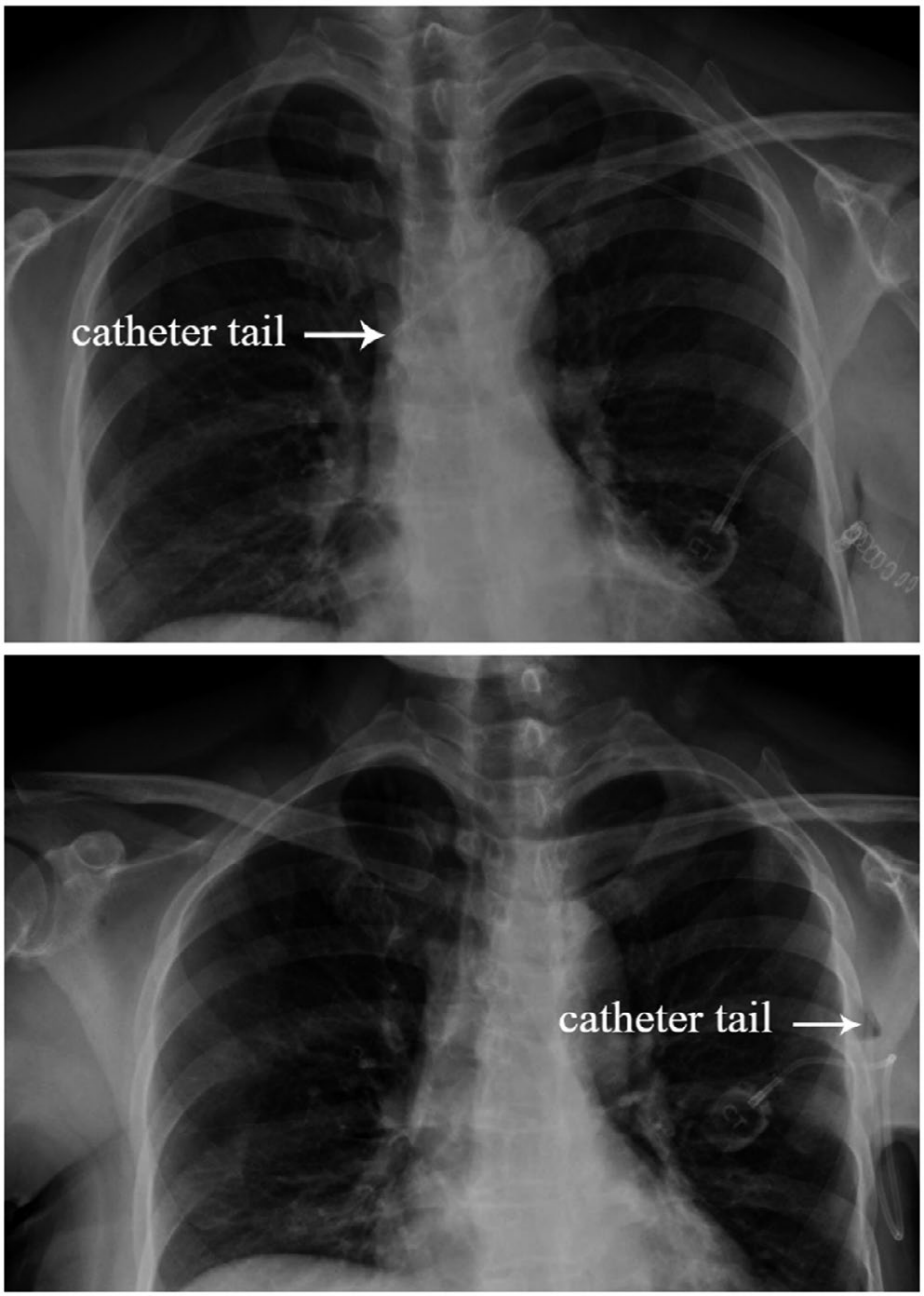
X ray results of catheter dislodgement

The intraoperative ipsilateral axillary PC implantation has some specific complication: catheter dislodgement from the vein. Due to the potential subcutaneous cavity of the operation area (axillary, thoracic wall) and the exercise of ipsilateral upper limb, the catheter could migrate and even drop out from the vein. No blood drainage from the port before use could be the clinical symptom for catheter dislodgement, which could be further confirmed by X‐ray (see Figure 3). In our study, 4 of 76 patients suffered from catheter dislodgement. Catheter dislodgement always happen in first 2–4 weeks after operation. When catheter dislodgement happened, the PC should be removed.

#### 3.2.4 Removal of PCs

**Figure 4:**
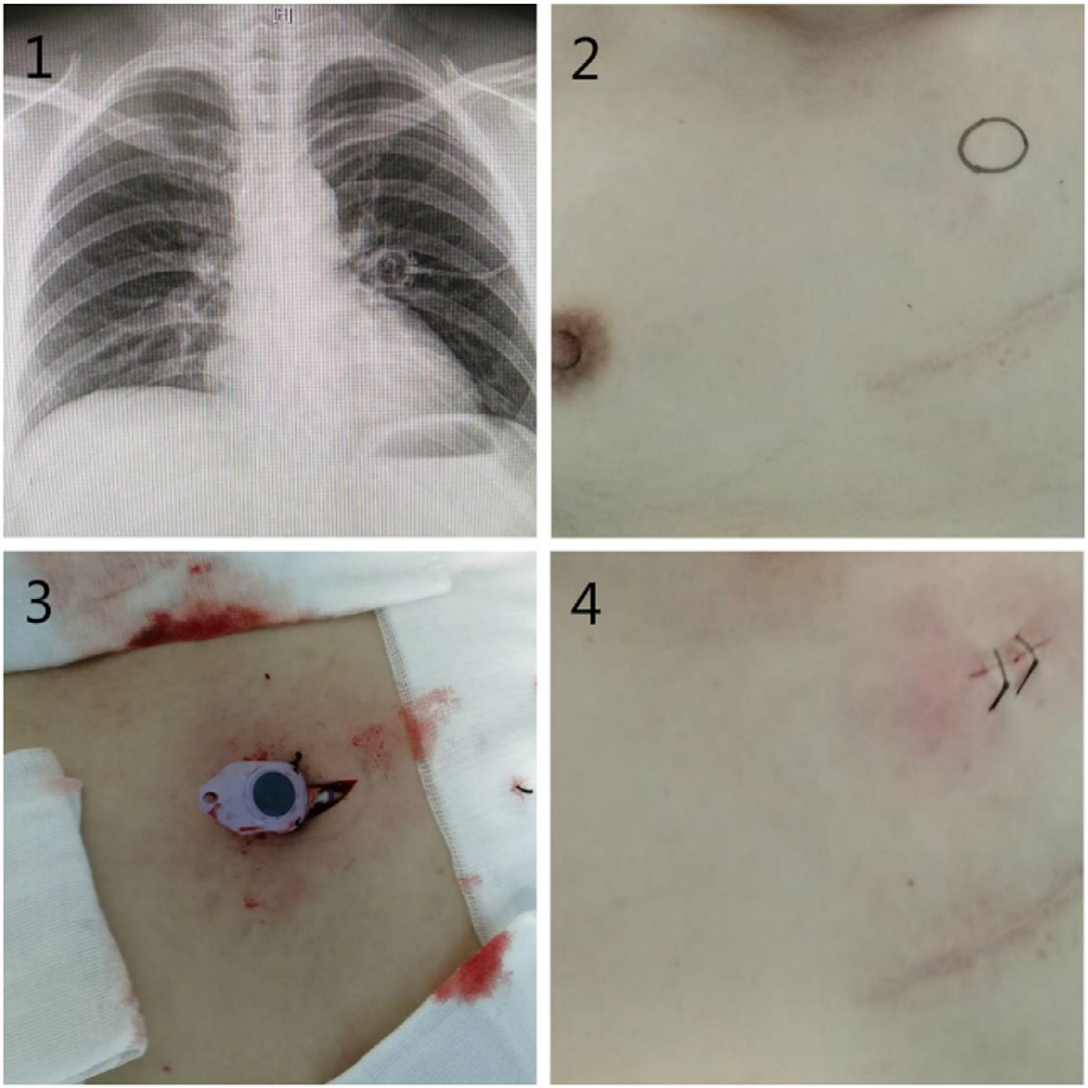
Removal of ipsilateral PCs under local anesthesia

Overall, PCs of eleven patients (10/76, 13.2%) have to been removed due to severe complications (6 VTE, 4 Catheter dislodgement). Most patients (66/76, 86.8%) with intraoperative ipsilateral axillary PC implantation have completed the whole chemotherapy successfully. Removal of PCs is performed under local anesthesia (see Figure 4).

### 3.3 Intraoperative ipsilateral axillary PC implantation gains more patients’ satisfaction

We use standardized questionnaires to evaluate the 30 patients’ satisfaction toward percutaneous conventional contralateral PC implantation. We figure out that the average scores for sensation of pain, level of tension, and their overall satisfaction are 4.3 points, 4.4 points, and 6.2 points, respectively. Due to the general anesthesia and shorter time consuming, intraoperative implantation gains significantly more favors in all aspects (no sensation of pain, no level of tension, 100% overall satisfaction).

## 4 DISCUSSION

Adjuvant chemotherapy plays an important role in the comprehensive treatment of breast cancer. In recent years, pre‐operative examinations have provided more and more information for clinicians in tailoring the individualized therapy, mostly due to pathological test of primary tumor and regional lymph nodes (axillary or internal mammary).^3^ The indications for adjuvant chemotherapy could be confirmed for majority of breast cancer patients before operation.

PICC and PC are the standard center venous devices for chemotherapy. In our institute, PC is more preferred choice, for its convenience in use: iterative perfusions without weekly maintenance. However, the insertion of PC is invasive, and the patients’ satisfaction during this procedure is an embarrassing problem. In our study, we have assessed these factors by standardized VAS questionnaires in patients receiving conventional PC insertion under local anesthesia. We figure out that patients’ satisfaction is significantly influenced by the anxiety and mental tension, and pain of invasive practice.

In our study, we explore and develop a SOP for ipsilateral axillary/subclavian PC implantation during breast cancer operation, which perfectly resolves the problems above for selected patients. Due to the general anesthesia and shorter time consuming, patients no longer suffer these embarrassing problems, thus, their satisfaction towards PC implantation have raised to a high extent. Moreover, thanks to the puncture of axillary/subclavian vein under direct eyesight of surgeons, the procedure of intraoperative PC implantation can shorten the time consuming significantly and avoid severe complications of puncture (hemathorax, pneumothorax, and puncture of artery) effectively.

As described in the Results part, the intraoperative subclavian PC implantation was convenient and took only about 11 min, this procedure did not increase the amount of general analgesics that patients demand. Moreover, adding this novel procedure in breast cancer operation did not extend the recovery time after operation, when compared with those patients just receiving regular operation (mean time for removing drainage tubes: 7.05 vs. 6.46 days). This indicates that intraoperative subclavian PC implantation would not increase the extra‐hospitalizations significantly. In our study, since both approaches of subclavian PC implantation are minimal invasive practices, we did not record the bleeding volume, which was not a regular concerning factor in the previous studies too.

In the previous literature, the major PC‐related complications are infection and VTE, which could lead to the removal of PC. The incidence for PC‐related infection is approximately 3–12%,^9^, ^25^ while that incidence for VTE is about 11–15% at 6 months.^14^, ^26^ In our study, our data show that incidence for major complications of intraoperative PC implantation is similar to conventional PC implantation.

Some traditional surgeons take the opinion that the veins of ipsilateral sick side should avoid blood drainage and central venous catheter insertion to aggravate lymphedema. However, in recent years, more and more studies demonstrated that there was no difference in port complications or lymphedema rates between patients who had ports placed on the ipsilateral side compared with the contralateral side for breast cancer treatment.^22^, ^27^

There are still some problems to be addressed further. First, in our study, there are about half of the patients with ipsilateral subclavian PC received radiotherapy. Whether radiotherapy would increase PC‐related complications is a real question, which needs more patients and further follow‐up to explore. Second, in the present study, we only choose and conduct ipsilateral subclavian PC implantation in patients receiving mastectomy and ALND. Whether this method could be applied in patients receiving breast conserving therapy or sentinel lymph node biopsy, need more time and practice. Third, the present study is not a rigorous randomized clinical trial (RCT). We admits that RCT is the best way to assess the safety and efficiency of a clinical intervention, however, since the technique is not a therapeutic treatment and the choice of PC implantation approach was at the discretion of surgeons, we conducted the present study as a prospective clinical research. Maybe in future RCT is needed to further explore the clinical significance of this technique.

## 5 CONCLUSION

In the present study, we develop a SOP for intraoperative ipsilateral axillary/subclavian PC implantation in resectable breast cancer patients, which is noval, convenient, and safe. In selected breast cancer patients with indications for adjuvant chemotherapy, this practice could significantly shorten the time consuming of PC implantation and improve the degree of patients’ satisfaction.

## CONFLICT OF INTEREST

The authors declare to have no competing interest.

## AUTHOR CONTRIBUTIONS

Conceptualization, funding acquisition, writing‐review, and editing: Feng Ye, Xiaoming Xie, and Jun Tang. Data curation, formal analysis, writing‐original draft: Feng Ye, Yubo Liu, and Ping Yu. Methodology, data curation: Yan Wang, Na Li, and Ping Yu. All the authors were all involved in approval of the final version.

## ETHICS APPROVAL AND CONSENT TO PARTICIPATE

All participants have signed informed consent and this study was approved by our Ethics Committee.

## Supporting information

Supplementary Material

Supplementary Material

## Data Availability

The data used to support the findings of this study are included within the article.
