# Supplementary material for: Intraoperative ipsilateral subclavian port catheter implantation in resectable breast cancer patients: A novel, safe, and convenient clinical practice
Source: Cancer Med. 2020 Nov 4;9(23):8970–8. doi: 10.1002/cam4.3595 (PMC7724495; doi:10.1002/cam4.3595)
Supplement: Supplementary file 1 — Supplementary Material [file CAM4-9-8970-s001.docx]

SOP for a conventional contralateral PC implantation under local anaesthesia (Supplementary Fig 1)

1. The patient is postured by supine position.The contralateral anterior chest and neck was prepared and draped. A circulating nurse was positioned near the patient’s head and constantly checked the patient’s condition during the operation. Patients were asked to turn their head to the other side. Conventional surgery area disinfection, shop sterile surgical towels.
2. An ECG monitor, a portable B ultrasonic instrument and a port catheter and accessories were prepared.
3. After local anesthesia, the surgeon inserted the introducer needle and did the subclavian vein puncture under the guidance of B ultrasonic instrument.
4. When the subclavian vein was accessed (see blood when gently withdrawing the plunger of the syringe), a J-tip guide wire is put in the vein through the introducer needle.
5. Then the syringe is removed and the dilater is put in. The guide wire is removed and the catheter is put in subclavian vein to a suitable depth (usually 15-20cm according to the height of patient).
6. After a second local anesthesia, a 3 cm transverse incision was made just 3-4 cm distal to the lower margin of the clavicle. Gentle dissection of the subcutaneous tissue was done with electrocautery until the fascia layer was reached. At this layer, a pocket for the port was created with blunt and sharp dissection.
7. The catheter is passed subcutaneously from the puncture site to the pocket by using the rod. The catheter is cut at an appropriate length (usually 19-24cm).
8. Surgeons link the catheter to the port and embeded the port in the pocket and fixed it with suture.
9. After checking the patency of the port and flushing it with heparin, the wound was closed. **Time consuming of implantation is recorded from step 2 to step 9.**
10. After returning from the operating room, a routine chest X-ray was obtained to check the catheter position and to rule out postoperative complications like pneumothorax.


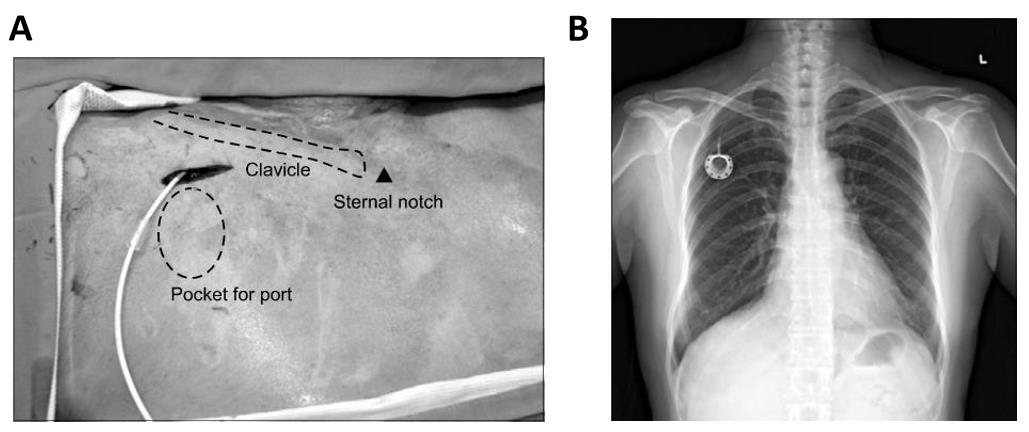


Supplementary Fig 1. SOP for a conventional contralateral subclavian PC implantation

1. sketch map of a conventional contralateral subclavian PC implantation
2. postoperative X-ray check of a conventional contralateral subclavian PC implantation

(Figure quoted from Keum DY. Korean J Thorac Cardiovasc Surg. 2013 Jun;46(3):202-7.)
